# β-Methylamino-L-alanine substitution of serine in SOD1 suggests a direct role in ALS etiology

**DOI:** 10.1371/journal.pcbi.1007225

**Published:** 2019-07-19

**Authors:** Elizabeth A. Proctor, David D. Mowrey, Nikolay V. Dokholyan

**Affiliations:** 1 Departments of Neurosurgery, Pharmacology, and Biomedical Engineering, The Pennsylvania State University, Hershey, Pennsylvania, United States of America; 2 Department of Biochemistry & Biophysics, University of North Carolina at Chapel Hill, Chapel Hill, North Carolina, United States of America; 3 Departments of Pharmacology, Biochemistry & Molecular Biology, Chemistry, and Biomedical Engineering, The Pennsylvania State University, Hershey, Pennsylvania, United States of America; Clemson University, UNITED STATES

## Abstract

Exposure to the environmental toxin β-methylamino-L-alanine (BMAA) is linked to amyotrophic lateral sclerosis (ALS), but its disease-promoting mechanism remains unknown. We propose that incorporation of BMAA into the ALS-linked protein Cu,Zn superoxide dismutase (SOD1) upon translation promotes protein misfolding and aggregation, which has been linked to ALS onset and progression. Using molecular simulation and predictive energetic computation, we demonstrate that substituting any serine with BMAA in SOD1 results in structural destabilization and aberrant dynamics, promoting neurotoxic SOD1 aggregation. We propose that translational incorporation of BMAA into SOD1 is directly responsible for its toxicity in neurodegeneration, and BMAA modification of SOD1 may serve as a biomarker of ALS.

## Author summary

The environmental toxin β-methylamino-L-alanine (BMAA) has been linked to cases of amyotrophic lateral sclerosis (ALS), but the role of this compound in disease is unknown. We propose that BMAA becomes incorporated into the ALS-linked protein Cu,Zn superoxide dismutase (SOD1), destabilizing it and promoting formation of the protein aggregates characteristic of ALS. Using computational techniques focused on the structure of BMAA-incorporated SOD1, we demonstrate that the presence of BMAA changes SOD1 structure and dynamics to promote aggregation. We propose that BMAA incorporation in SOD1 in the mechanism of the compound’s link to ALS, and that BMAA modification may serve as a biomarker for environmentally-linked cases of ALS.

## Introduction

Amyotrophic lateral sclerosis (ALS) is a motor neurodegenerative disease that affects 2–9 individuals per 100,000 every year [1]. More than 150 mutations to Cu,Zn superoxide dismutase (SOD1) have been associated with ALS. Misfolded and aggregated SOD1 has been found in motor neurons in both sporadic and familial ALS [2]. In recent studies, a non-native trimeric oligomer of SOD1 has been shown to be toxic in the hybridized motor neuron cell line NSC-34, suggesting a causative role of misfolded SOD1 aggregates in ALS etiology [3]. The phenomenon of SOD1 misfolding is puzzling due to the protein’s remarkable stability (ΔΔG >20 kcal/mol) [4]; the mild destabilization (<5 kcal/mol) caused by ALS-linked mutations [5] does not significantly reduce the stability of SOD1 from that of the average human protein (~5–15 kcal/mol [6,7]), and so does not explain SOD1 misfolding [8,9]. Previous studies have demonstrated that post-translational modifications of SOD1 can contribute to destabilization [10], and that glutathionylation of Cys111 promotes SOD1 dimer dissociation, the required initial step for SOD1 aggregation [11], by ~1,000 fold [12,13]. Environmental toxins that modify proteins have also been proposed to play a role in ALS etiology.

The indigenous Chamorro population on Guam have an ALS incidence 100 times larger than the worldwide average, which has been linked to an enrichment of the toxin β-methylamino-L-alanine (BMAA) in their diet [14]. The quest for the mechanism of BMAA toxicity resulted in the hypothesis that this amino acid is misincorporated into proteins [15], resulting in formation of inclusion bodies in neurons [16]. Studies have demonstrated synergistic toxicity of ALS-linked mutant SOD1 and BMAA [17], yet no reports of misincorporation have been presented. A large-scale proteomic study has identified multiple proteins that featured misincorporated BMAA [18]. However, the reported misincorporation rates were low. Despite the low misincorporation rates, Ackerman and colleagues have argued that even a rate of 1 misincorporation per 10,000 codons can lead to neurodegeneration in mice [19]. Hence, identification of BMAA misincorporation into SOD1 may have been overlooked due to sensitivity issues, and never reported.

We propose that misincorporation of BMAA into SOD1 destabilizes the protein, increases aggregation propensity, and thus promotes ALS onset and progression. We hypothesize that BMAA can directly modify SOD1 by incorporation in place of serine during translation. As a proof of principle, we perform a computational analysis predicting the effects on thermodynamic stability of substituting BMAA in place of each of the ten serines in SOD1. We find remarkable destabilization of SOD1 due to BMAA misincorporation at all sites, strongly suggesting a direct role of this toxin on the etiology of ALS. We perform molecular dynamics simulations of modified SOD1^S107B^ to evaluate the structural impact of such substitution, and find significant dynamic changes to residues participating in metal-binding and the intra-monomer disulfide bond, key structural determinants of SOD1 stability. These findings suggest a mechanism for the toxicity of BMAA in ALS, and provide support for the candidacy of BMAA as a long-sought biomarker for ALS.

## Results

### SOD1 serine to BMAA mutation destabilizes SOD1 dimers

We evaluate the effects of replacing serine residues with BMAA in the SOD1 dimer. Because misincorporation is a rare event, more than one instance in the same molecule would be unlikely, and thus we study the scenario of BMAA misincoporation into only one monomer of the SOD1 heterodimer. We computationally substitute each individual serine residue in SOD1 (PDB ID: 1SPD) to BMAA, and estimate the resulting changes in free energy (ΔΔG) of the structure. To control for the effect of computational mutation, we also perform the same calculation while converting the given residue to lysine. Lysine, similar to BMAA, is also an unbranched, positively-charged amino acid. We find that while mutations of each serine to either BMAA or lysine generally destabilize the SOD1 dimer (Table 1), mutations to BMAA result in significant destabilization, while mutations to lysine result in minor (<2 kcal/mol) or negligible (<1 kcal/mol) destabilization, and in some cases ΔΔG is within error of zero. We conclude from these results that substitution of BMAA for serine in the SOD1 structure results in an unfavorable structural shift resulting in thermodynamic destabilization, likely due to steric effects from the larger BMAA molecule.

**Table 1:** SOD1 Serine to BMAA mutation destabilizes SOD1 dimers. Folding free energy differences between wild type and mutant SOD1, ΔΔG. Values are mean ± standard deviation among 20 independent runs. Calculations are performed using *Eris* with both fixed and flexible backbone algorithms for serine to BMAA, and to lysine, for comparison.

Mutation	ΔΔG, kcal/mol	
	BMAA	LYS
S25	3.1 ± 0.2	1.9 ± 0.2
S34	3.7 ± 0.4	0.2 ± 0.5
S59	6.9 ± 0.4	0.7 ± 0.4
S68	4.7 ± 0.4	1.9 ± 0.4
S98	3.4 ± 0.2	0.5 ± 0.2
S102	3.9 ± 0.5	-0.1 ± 0.4
S105	6.4 ± 0.5	-0.5 ± 0.6
S107	3.1 ± 0.6	0.8 ± 0.4
S134	4.3 ± 0.6	-0.2 ± 0.6
S142	3.5 ± 0.3	1.4 ± 0.4

### Thermodynamic destabilization of SOD1 by modification with BMAA

**Fig 1:**
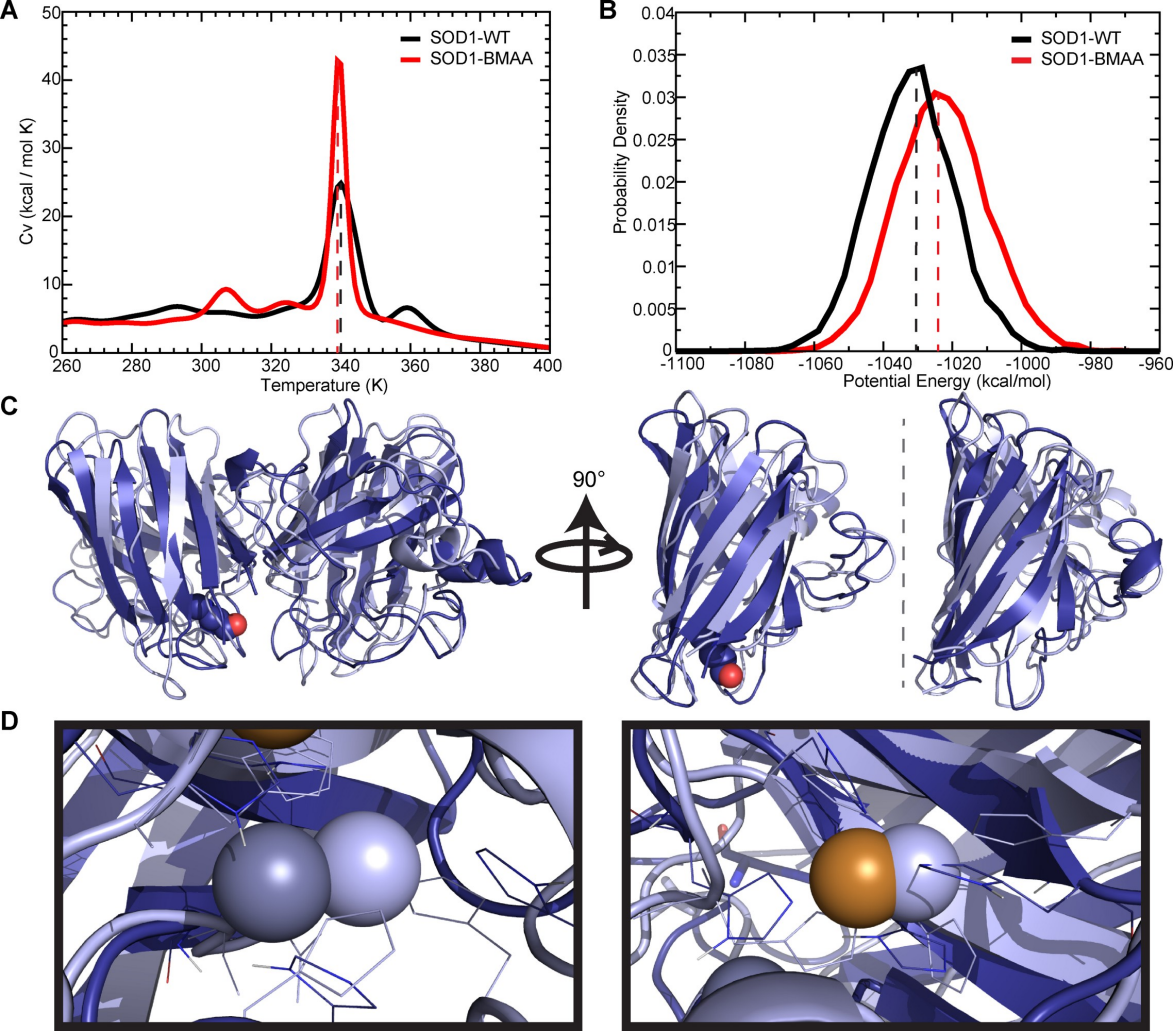
BMAA modification induces thermodynamic destabilization and structural changes in SOD1. (A) Specific heat curves generated from replica exchange DMD simulations of BMAA-modified or wild type SOD1. Peaks in specific heat indicate melting events. Dotted lines indicate major melting event for each species. (B) Histogram of potential energy of BMAA-modified or wild type SOD1 gathered from single(low)-temperature DMD simulations. SOD1-BMAA exists at a mean higher potential energy than wild type SOD1, indicating a less favorable structural conformation. Dotted lines indicate peaks. (C) Structural alignment of BMAA-modified (dark blue) and wild type (light blue) structures. Root mean square distance (RMSD) between structures is 3.24 Å. The β-strands in the dimer interface (right, cutaway of each monomer viewed from the center of the dimer interface) can be seen to be elongated and twisted in BMAA-modified SOD1 (dark blue) as compared to wild type (light blue). BMAA is shown as spheres. (D) Structural alignment of Zn- (left) and Cu- (right) binding sites of BMAA-modified (dark blue) and wild type (light blue) structures. Ions belonging to the BMAA-modified structure are in color (dark grey for Zn, orange for Cu), while ions belonging to the wild type are in light blue. Metal-coordinating residues for both structures are shown as lines.

To obtain the thermodynamic melting curve of BMAA-SOD1, we perform replica exchange DMD simulations at a wide range of temperatures. As a demonstration of potential effects of BMAA, we choose substitution of S107, as the smallest predicted ΔΔG (Table 1) upon misincorporation of BMAA. Misincorporation of BMAA at a site with a larger predicted ΔΔG would be likely to have larger thermodynamic effects. We find that the incorporation of BMAA into the SOD1 structure in place of serine-107 shifts the melting temperature of the protein by only ~2°C (Fig 1A). However, we observe evidence of lower temperature localized unfolding events present in BMAA-SOD1 that are absent from the unfolding of WT-SOD1, which displays one dominant peak in C_V_ representing coupled dimer dissociation and monomer unfolding [13]. Supporting this hypothesis, we find that BMAA modification increases the potential free energy of the low-energy “ground state” of the SOD1 dimer, decreasing the stability of the native state (Fig 1B). This destabilization makes BMAA-SOD1 more likely to undergo localized unfolding events that can expose toxic epitopes, as well as lead to the protein aggregation characteristic of ALS. This destabilization of the SOD1 dimer by BMAA substitution provides a mechanism for the linkage of BMAA poisoning to ALS etiology.

### SOD1 structural changes induced by modification with BMAA

To test our conclusion that mutation of serine to BMAA results in a significant structural change in SOD1, we perform discrete molecular dynamics (DMD) simulations of SOD1 with BMAA incorporated into one monomer of the structure in place of Ser107, the site at which BMAA misincorporation was predicted to have the smallest thermodynamic effect. Misincorporation of BMAA at a site with a larger predicted ΔΔG (Table 1) would be likely to have larger structural changes. Upon building and equilibrating our model of BMAA-SOD1, we find rearrangement of the beta-barrel of the modified monomer, and resulting lengthening and twisting of the beta-strands that form the SOD1 dimer interface (Fig 1C), with a total root mean square structural deviation of 3.24 Å. Although metal ions are necessarily constrained to their ligands in our simulations, we note that the distortion of the SOD1 structure extends to shifts in the orientation of metal-binding residues, especially those coordinating Zn (Fig 1D), which would potentially affect the binding affinity of Cu and Zn *in vitro* and *in vivo*. Binding of metal ions, especially Zn, contributes significantly to the stability of SOD1, and destabilization and loss of bound metal ions is the second step in SOD1 aggregation [11], and metal-binding residues feature several known ALS-linked mutations.

### BMAA-SOD1 features dynamically destabilized disulfide bond and metal ion binding sites

**Fig 2:**
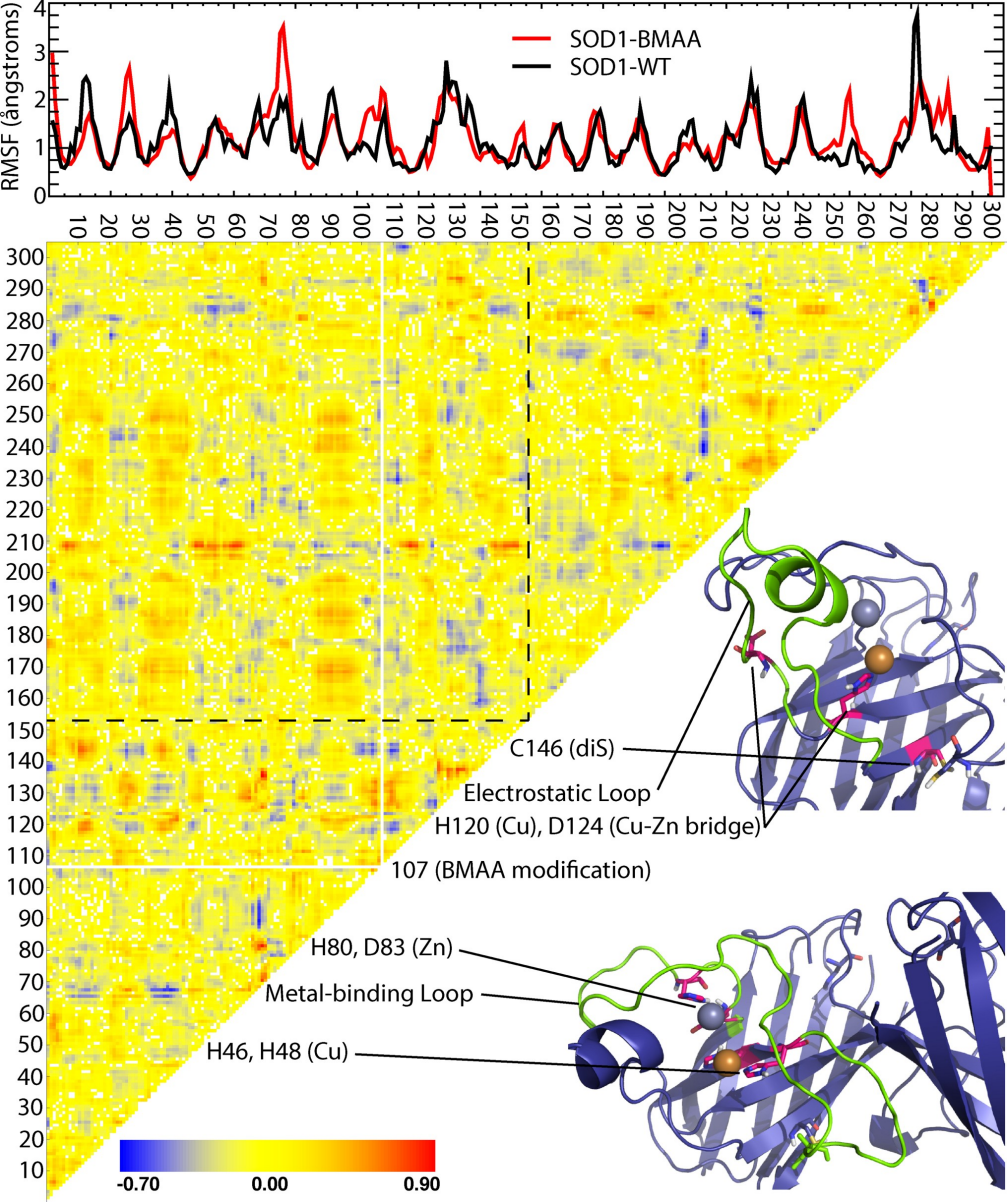
BMAA-SOD1 features altered dynamics near disulfide bond and metal ion binding sites. Heat map of changes in correlated motions between residues (standard sequence numbering provided) upon modification with BMAA. Red indicates increased correlated motions, blue indicates decreased correlated motions, and yellow indicates no change. Dotted line indicates the divide between the two monomer chains. Select significantly affected structural features critical for SOD1 stability are labeled, with location on the SOD1 structure highlighted. A comparison of root mean square fluctuations (RMSF) at each residue for SOD1 with and without BMAA modification is included (top), highlighting regions of increased or decreased flexibility due to misincorporation of BMAA.

To further investigate the potential effects of incorporation of BMAA into the SOD1 structure, we analyze the dynamics of the BMAA-modified protein in low-temperature steady-state simulations and compare with wild-type protein. Changes in root mean square fluctuation (RMSF) over the length of the protein (Fig 2, top) upon BMAA modification reveal increased flexibility in the metal-binding loop (residues 49–84) and the residues directly surrounding the BMAA modification, as well as flexibility differences caused by slight shifts in the residues included in β-strands 1, 2, and 3 due to the rearrangement in β-barrels discussed above.

While changes in RMSF indicate differences in local stability, correlated dynamics are a more informative measure of the effect of protein modification on overall structure, stability, and function because they reveal dynamic coupling between distal regions of the protein [20]. Changes in dynamic coupling across SOD1 due to BMAA misincorporation would change not only local stability, but also how local instabilities are propagated to other regions of the protein, potentially resulting in additional changes to structurally important features. In calculating the correlated motions of residue pairs [20], we find profound differences in the motions of residues corresponding to key structural features of SOD1 known to promote integrity of the properly folded structure (Fig 2): namely, both cysteines of the intra-monomer Cys57-Cys146; the Cu-binding histidines 46, 48, and 120; the Cu-Zn bridging ligand His63; the Zn-coordinating residues His71, His80, and Asp83; and the structurally important residue Asp124, which forms a crucial connection between Cu- and Zn- binding residues and whose mutation has been linked to ALS [21]. We also observe significant disturbances to large portions of both the electrostatic loop and the metal-binding loop, which contribute to enzymatic function, maintain structural integrity, coordinate the binding of the metal ions, and prevent protein aggregation [22]. Together, these findings strongly support the conclusion that the incorporation of BMAA into SOD1 causes both static and dynamic structural disturbances that result in local destabilization of the region surrounding the modification, including the nearby electrostatic loop, and propagation of those instabilities to important structural features of the protein, leading to increased propensity for misfolding and aggregation. This work supports an SOD1-linked mechanism for the toxicity of BMAA in environmentally caused cases of ALS.

## Discussion

SOD1 dimer dissociation has been shown to be the first step in the misfolding and aggregation of SOD1 [11]. Proctor *et al*. [3] recently demonstrated that the association of misfolded SOD1 monomers into a non-native trimeric oligomer results in cytotoxicity in hybridized motor neurons. The remarkable thermodynamic stability of unmodified wild type SOD1 protects against this first necessary step of dimer dissociation [5], thus also protecting against the formation of toxic oligomers. However, the addition of exogenous factors to the SOD1 structure, such as post-translational modifications, has been shown to have a profound destabilizing effect on dimer stability [10,12,23]; oxidative glutathionylation is a particularly severe example of such a modification [12,24]. Given the high fraction (90%) of sporadic ALS cases as compared to those with a known genetic link, we have long hypothesized that other post-translational modifications may similarly impact SOD1 stability. BMAA is a good candidate because, while not overly abundant, this cyanobacteria-produced neurotoxin has been linked to significantly increased occurrence of sporadic ALS in populations with frequent dietary consumption of food sources containing high levels of BMAA [14,16].

**Fig 3:**
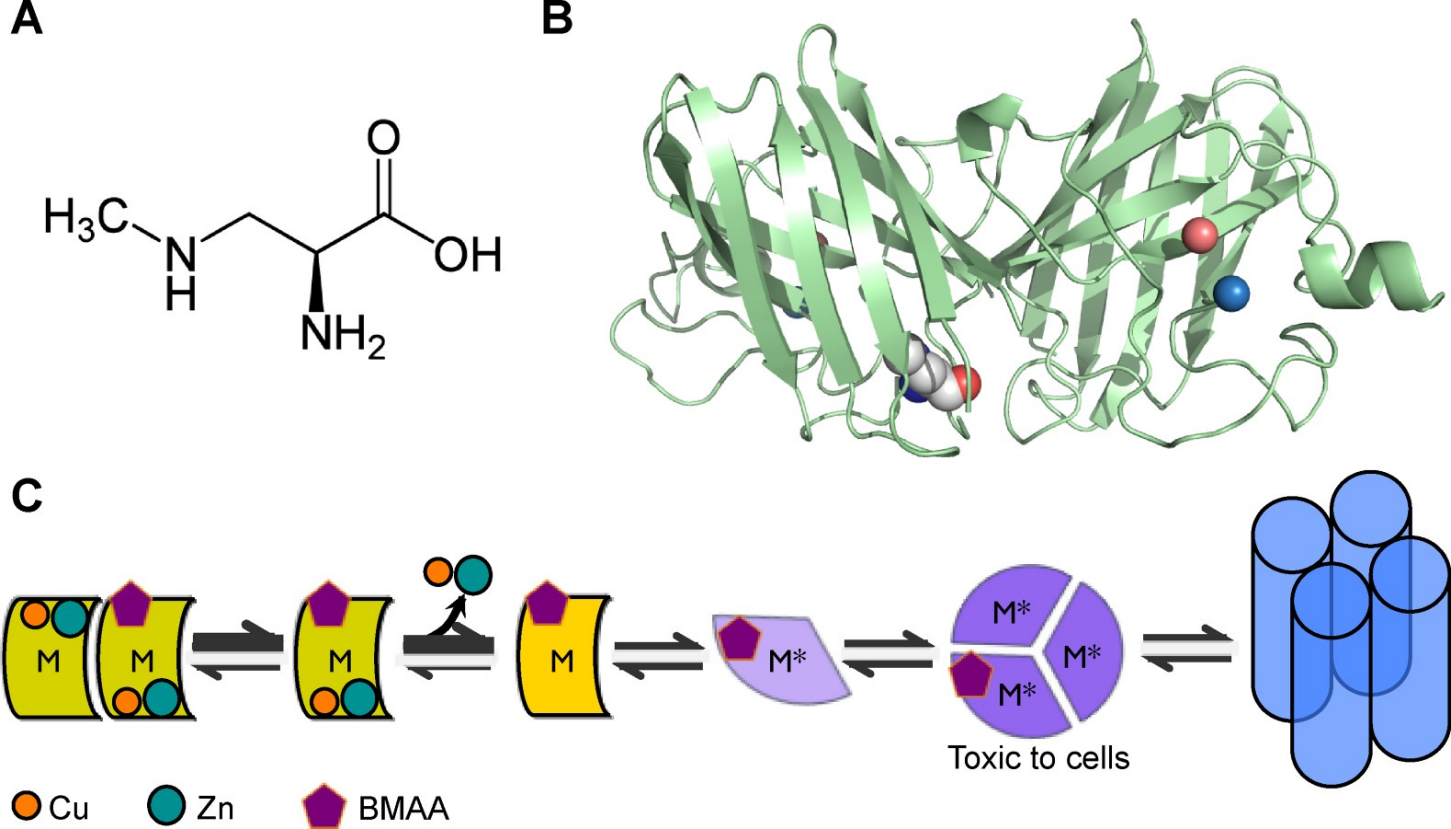
Proposed mechanism of BMAA toxicity in ALS pathology. (A) Chemical structure of BMAA molecule. (B) Misincorporation of BMAA for serine causes structural rearrangement and strain that propagates to the dimer interface and metal-binding residues. BMAA is show as spheres colored by atom type; copper (orange) and zinc (cerulean) ions are shown as spheres. (C) From left to right: misincorporation of BMAA into SOD1 promotes dimer dissociation and destabilization of the metal-binding sites; metal binding is further destabilized in the monomeric form, leading to metal loss; without metal ions, the SOD1 monomer fold is destabilized misfolds; misfolding promotes oligomerization and the formation of non-native SOD1 trimer, previously shown to be neurotoxic; misfolded SOD1 monomer can also form fibrils observed in ALS patients.

In this work, we present the hypothesis, based on others’ experimental and epidemiological observations [15,18], that BMAA can be incorporated into SOD1, and demonstrate using computational structural analysis and simulation that incorporation of BMAA would promote SOD1 dissociation, loss of metals, and misfolding. Misfolded SOD1 then aggregates to form oligomers that, through as yet unknown mechanisms result in motor neuron death, thereby contributing to the neurotoxicity of BMAA and its linkage to sporadic ALS in areas of environmental contamination (Fig 3). We speculate that BMAA incorporation into SOD1 may be rare, explaining why this modification has not yet been reported. However, even rare events may promote an avalanche of misfolding events; the initiating destabilization by BMAA incorporation may serve as a nucleating event for the misfolding and aggregation of SOD1 through the templating mechanism [25–29]. Our analysis suggests the need for a comprehensive study of SOD1 modification patterns in ALS patients in order to uncover mechanistic patterns of disease onset and progression, and aid in understanding of potential lifestyle and preventative interventions for sporadic ALS.

## Methods

### ΔΔG calculations

We determine the changes in free energy (ΔΔG) for mutations of each serine residue in the SOD1 dimer (PDB ID: 1SPD) to β-methylamino-L-alanine (BMAA) or lysine using *Eris* [30,31]. Reported ΔΔG values represent the mean ± standard deviation of 20 independent rounds of *Eris* calculation. Each round of *Eris* calculation produces an expected value of the ΔΔG of mutation from 20 independent simulations for both wild-type and mutant protein with each simulation consisting of 20 steps of Monte Carlo optimization.

### BMAA parameterization

The BMAA rotamer library was generated using the Rosetta MakeRotLib protocol [32]. We used the Gaussian 09 program (Gaussian, Inc.) to optimize the initial structure of BMAA at the HF 6-31G(d) level of theory with a polarized continuum model of the aqueous solvent, which appropriately shields the positive charge on the BMAA side chain. We generate a backbone-dependent rotamer library from the initial structure using 10° increments for both φ and ψ angles for a total of 1296 (= 36 × 36) φ/ψ bins, within which each of the two χ angles of BMAA were sampled at 30° increments. The MakeRotLib protocol was used to obtain mean angles and probabilities for all combinations of the three staggered conformations for the two χ angles in each φ/ψ bin. Lysine parameters for Ramachandran probabilities, χ angle standard deviations, and the reference energy were used for both BMAA and lysine, as both feature unbranched, positively-charged side chains. The residue type parameter file for BMAA was built using pre-existing atom types in the CHARMM-based Medusa force field [33].

### All-atom discrete molecular dynamics

DMD implements step function potentials to describe inter-atomic interactions, as opposed to the continuous potentials used in traditional molecular dynamics (MD) [34–36]. We utilize an all-atom protein model that explicitly represents all heavy atoms and polar hydrogen atoms. Bonded interactions are represented using infinite square-well constraints for bond lengths, bond angles, and dihedral angles. Non-bonded interactions are adapted from the continuous CHARMM-based Medusa force field [33], van der Waals interactions are modeled using the Lennard-Jones potential, and solvation interactions are modeled using Lazaridis-Karplus solvation [37], all discretized by multi-step square-well functions for use in DMD. We model hydrogen bonding interactions using the reaction algorithm [38]. The DMD simulation engine (πDMD, v1.0) with Medusa all-atom force field is available from Molecules In Action, LLC (free to academic users, moleculesinaction.com).

### Modeling of BMAA-SOD1

Using the known X-ray crystallographic structure of wild type SOD1 (PDB ID 1SPD) as a reference structure, we deleted serine 107 from one monomer and replaced it with BMAA, which was joined in the peptide chain of SOD1 using peptide bond constraints and equilibrated using the discretized Medusa force field [33] in DMD with an iterative relaxation and equilibration protocol as previously described [13].

### Replica exchange

We use the replica exchange method to construct a thermodynamic profile of BMAA-SOD1 unfolding [39]. Independent replicas of the simulation system of interest are run in parallel at 16 different temperatures: 0.48 (∼242 K), 0.495 (∼ 249 K), 0.51 (∼ 257 K), 0.525 (∼ 264 K), 0.54 (∼ 272 K), 0.555 (∼ 280 K), 0.57 (∼ 287 K), 0.585 (∼ 295 K), 0.60 (∼ 302 K), 0.615 (∼ 310 K), 0.63 (∼ 317 K), 0.645 (∼ 325 K), 0.65 (∼ 327 K), 0.67 (∼ 337 K), 0.69 (∼ 347 K) and 0.71 (∼357 K) kcal (mol kB)^–1^. Every 50 ps, replicas neighboring in temperature attempt to exchange temperature values according to the Metropolis criterion. The replica exchange method increases sampling efficiency by allowing energetic barriers to be overcome with exposure to higher temperatures. We note that temperatures used in MD simulations do not directly equate to physical temperatures, but are useful to evaluate relative differences between systems.

### WHAM analysis of C_V_

Replica trajectories were combined for the analysis of folding thermodynamics using the MMTSB tool [40] for weighted histogram analysis method (WHAM) [41]. WHAM computes the density of states by combining energy histograms from simulation trajectories with overlapping energies and calculates the folding specific heat at constant volume at a function of temperature.
